# Simultaneous reconstruction of attenuation and activity in ToF PET/MRI with additional transmission data

**DOI:** 10.1186/2197-7364-2-S1-A33

**Published:** 2015-05-18

**Authors:** Ester D’Hoe, Pieter Mollet, Ekaterina Mikhaylova, Michel Defrise, Stefaan Vandenberghe

**Affiliations:** MEDISIP Medical Imaging and Signal Processing Group, Ghent University, IBBT-IBiTech, iMinds Medical IT, Ghent, Belgium; Department of Nuclear Medicine, Vrije Universiteit Brussel, Brussels, Belgium

In Time-of-Flight PET/MRI systems accurate attenuation correction, based on the MRI image, is not straight forward. An alternative is attenuation correction based on emission data only. This is for instance done by simultaneous reconstruction of attenuation and activity with the MLAA algorithm, but the method as originally proposed has certain limits. The attenuation can only be determined up to a constant and in regions of low tracer uptake, the method results in less accurate attenuation values. An adapted MLAA algorithm has been proposed to overcome this issues and was successfully applied on simulation studies. The so called MLAA+ algorithm uses regular PET emission data as well as transmission data. This transmission data is acquired after insertion of an annulus shaped transmission source into the scanner bore. The Time-of-Flight information allows to separate transmission and emission data in a simultaneous acquisition. With the transmission data, an MLTR-based reference attenuation image is reconstructed. Afterwards, this attenuation image is used in the MLAA+ simultaneous reconstruction of attenuation and emission as a reference. We here propose the results of the reconstruction of patient data, based on the MLAA+ algorithm. In total, seven patients were scanned in a sequential PET/MRI scanner and afterwards in a CT scanner. The CT scan is used as an attenuation map to reconstruct the PET emission data with the well established MLEM algorithm. This reconstruction can be seen as the gold standard to which we can compare the MLAA and MLAA+ reconstructions. A preliminary study on one patient indicates that the MLAA+ algorithm results in better reconstructed emission and attenuation images as compared to the MLAA algorithm. If we compare the MLAA+ method to the gold standard, there is still room for improvement.

